# Assessing Visual Statistical Learning in Early-School-Aged Children: The Usefulness of an Online Reaction Time Measure

**DOI:** 10.3389/fpsyg.2019.02051

**Published:** 2019-09-13

**Authors:** Merel van Witteloostuijn, Imme Lammertink, Paul Boersma, Frank Wijnen, Judith Rispens

**Affiliations:** ^1^Amsterdam Center for Language and Communication, University of Amsterdam, Amsterdam, Netherlands; ^2^Utrecht Institute of Linguistics OTS, Utrecht University, Utrecht, Netherlands

**Keywords:** statistical learning, visual, online measure, reaction time, children

## Abstract

Visual statistical learning (VSL) was traditionally tested through offline two-alternative forced choice (2-AFC) questions. More recently, online reaction time (RT) measures and alternative offline question types have been developed to further investigate learning during exposure and more adequately assess individual differences in adults (Siegelman et al., 2017b, 2018). We assessed the usefulness of these measures for investigating VSL in early-school-aged children. Secondarily, we examined the effect of introducing a cover task, potentially affecting attention, on children’s VSL performance. Fifty-three children (aged 5–8 years) performed a self-paced VSL task containing triplets, in which participants determine the presentation speed and RTs to each stimulus are recorded. Half of the participants performed a cover task, while the other half did not. Online sensitivity to the statistical structure was measured by contrasting RTs to unpredictable versus predictable elements. Subsequently, participants completed 2-AFC (*choose correct triplet*) and 3-AFC (*fill blank to complete triplet*) offline questions. RTs were significantly longer for unpredictable than predictable elements, so we conclude that early-school-aged children are sensitive to the statistical structure during exposure, and that the RT task can measure that. We found no evidence as to whether children can perform above chance on offline 2-AFC or 3-AFC questions, or whether the cover task affects children’s VSL performance. These results show the feasibility of using an online RT task when assessing VSL in early-school-aged children. This task therefore seems suitable for future studies that aim to investigate VSL across development or in clinical populations, perhaps together with behavioral tasks.

## Introduction

Research into statistical learning (SL) has shown that infants, adults, and children are able to detect statistical structure in sequences of stimuli in the world around them (e.g., Saffran et al., 1996; Fiser and Aslin, 2002; Arciuli and Simpson, 2011, 2012). Extracting statistical properties from the input is thought to be an implicit process (Perruchet and Pacton, 2006) and has been observed in both the auditory (e.g., Saffran et al., 1996) and visual modalities (e.g., Kirkham et al., 2002; Conway et al., 2011), which has led to the suggestion that SL is a domain-general learning mechanism [see Frost et al. (2015) for a review]. SL has been put forward as an essential mechanism in language acquisition, which is supported by findings that have established relationships between an individual’s capacity for this type of learning and his/her language and literacy proficiency (e.g., Evans et al., 2009; Arciuli and Simpson, 2012).

In the typical SL paradigm, as originally employed by Saffran et al. (1996), participants are exposed to a continuous stream of visual or auditory stimuli (the *familiarization phase*). Without the participants’ knowledge, the stimulus sequences are divided into triplets of co-occurring elements (e.g., the continuous string *bidakupadotigolabu* is a concatenation of three-syllable chunks/triplets *bidaku*, *padoti*, and *golabu*). The order in which these triplets occur is free. Hence, transitional probabilities (TPs) are structured such that TPs from one syllable to the next are higher for stimuli within a triplet (e.g., *daku*) than for those that span a triplet boundary (e.g., *kupa*). It is crucial that during the familiarization phase, participants are not instructed to learn or memorize the input: they either listen passively or perform a cover task that is unrelated to the statistical regularities presented to them (e.g., Arciuli and Simpson, 2011). Under these task conditions, it is assumed that the learning process is implicit.

Participants were traditionally tested on their newly acquired knowledge of the TP structure in an *offline test phase*, subsequent to the familiarization phase. Such an offline test traditionally employed two-alternative forced-choice (2-AFC) questions, in which participants are presented with one group of three-syllable stimuli that co-occurred frequently during familiarization (e.g., the probable “word” *bidaku*) and one group of three-syllable stimuli that did not co-occur frequently (e.g., the less probable “non-word” *dakupa*). Whereas for infants the offline test phase consists of collecting listening or looking times, which are used to infer a preference for either familiar (word) or unfamiliar (non-word) items, adults and children can be asked explicitly which of the two patterns of stimuli is more familiar. In the latter case, above-chance performance on the group level is taken as evidence that participants have learned the contrast between the two patterns of stimuli, taken to reflect sensitivity to the TP structure presented to them during the familiarization phase. Bertels et al. (2012, 2015) showed that both adults and 9–12-year-old children who reach above-chance performance on an offline test phase had some degree of explicit knowledge of the TP structure as evidenced by confidence ratings (i.e., more confident in correct than incorrect items). Thus, although the learning process itself may be implicit, the resulting knowledge may (to some degree) be explicit.

The suitability of using offline 2-AFC questions for measuring SL has been questioned, especially for use in an individual differences approach (e.g., Siegelman and Frost, 2015; Kidd et al., 2017; Siegelman et al., 2017b). Furthermore, Siegelman et al. (2018) argue that offline measures inform us about the *learning outcome*, but do not reveal anything about the *learning process* during the familiarization phase. Conceivably, different individuals or different populations achieve similar offline performance, but these similar performances may be the result of differing learning trajectories during familiarization (Siegelman et al., 2018). Moreover, the term “SL” implies a temporal component: the assumption is that participants become increasingly responsive to the statistical structure during exposure. As explained by Batterink and Paller (2017), the initial stages of SL involve the encoding of the stimuli, which gradually transforms from the encoding of individual stimuli (e.g., syllables such as *bi*, *da*, and *ku*) to the encoding of larger co-occurring units (e.g., words such as *bidaku*). This development across time indicates increased sensitivity to the structure of the sequence. Analogously, learning during familiarization will increasingly allow participants to predict upcoming stimuli, resulting in faster reaction times (RTs) to predictable stimuli compared to unpredictable stimuli (Siegelman et al., 2018). This idea is based on the serial RT (SRT) task (Nissen and Bullemer, 1987), which measures participants’ implicit learning of a visuo-motoric sequence as the increase in RT when participants move from structured to unstructured, and thus from predictable to unpredictable, input.

Recent studies have employed the above-mentioned ideas about online learning in novel measures of SL with adult participants, providing insight into the initial and central stages of learning in adult learners, which are not tapped by offline measures (e.g., Misyak et al., 2010; Gómez et al., 2011; Karuza et al., 2014; Franco et al., 2015; Siegelman et al., 2018). The main aim of the present study is to extend these recent findings to child participants and to investigate the effectiveness of such an online measure with early-school-aged children, as previous studies employing online measures have focused on adult participants. Although several studies have shown children’s sensitivity to statistical structure in visual stimuli (e.g., Arciuli and Simpson, 2011, 2012; Conway et al., 2011), studies combining the use of on- and offline measures during such a task are scarce (but see Qi et al., 2018). Therefore, we adopted an online RT measure of the visual SL (VSL) paradigm, as developed by Siegelman et al. (2018), and assessed children’s learning through this measure. The development of online measures is especially important for studies investigating SL in early-school-aged children due to the fact that the traditional 2-AFC questions require explicit decision-making, a skill that young children have difficulties with (Bialystok, 1986). Children’s performance on 2-AFC questions in VSL tasks is known to increase between the ages of 5 and 12 years (Arciuli and Simpson, 2011; Raviv and Arnon, 2017; Shufaniya and Arnon, 2018). For this reason, solely using 2-AFC questions to assess early-school-aged children’s performance may not provide a complete picture of their SL abilities. In addition to the (implicit) online RT measure, we used two distinct (explicit) offline question types (2-AFC and 3-AFC) to investigate the usefulness of these measures with early-school-aged children. In the 3-AFC questions, participants do not choose the correct answer out of two as in traditional 2-AFC tasks, but complete a pattern by choosing the missing stimulus out of three alternatives (see e.g., Bertels et al., 2012, 2015; Siegelman et al., 2017b). Although this question type requires the participant to make an explicit judgment just as the 2-AFC questions, we hope that the 3-AFC questions are more intuitive for children and may therefore better reflect their SL abilities. Before turning to our methodology and results, we will present an overview of previous studies that have adopted online measures of SL.

### Online Measures of Statistical Learning

The most well-known task tapping SL abilities through an online measure is the SRT task (Nissen and Bullemer, 1987). Whereas the SRT is informative regarding the domain of visuo-motoric sequence learning, the fine motor skills implied in this task make it less suitable for use with certain participant groups known to have less developed fine motor skills [e.g., participants with specific language impairment and/or dyslexia (Hill, 2001; Ramus et al., 2003)]. Moreover, learning in the SRT task likely partially reflects sensitivity to a repeated sequence of movements, rather than pure sensitivity to statistical structure in (visual) stimuli (see e.g., Robertson, 2007; West et al., 2019). Tasks that have been employed to investigate other types of SL have largely focused on the use of offline measures of learning [e.g., VSL, artificial grammar learning, and non-adjacent dependency learning (NADL) tasks]. To further our understanding of the online SL process, both behavioral methods such as RTs and neurophysiological methods such as Electroencephalography (EEG) have been proposed as suitable online methods of investigating the learning trajectory of these alternative SL tasks. Although EEG has successfully been used to study SRT and artificial grammar learning tasks (for a review, see Daltrozzo and Conway, 2014), and has recently been applied to an auditory SL task similar to the one described above (Batterink and Paller, 2017), we focus here on behavioral methods employing RT-based measures of learning.

In 2010, Misyak et al. (2010) developed an online measure of SL that combined exposure to an artificial grammar containing non-adjacent dependencies with features of the classic SRT task. The grammar consisted of strings of the form *aXb*, where element *a* predicts element *b* (i.e., the non-adjacent dependency) and element *X* is a variable. Adult participants were exposed to an auditory speech stream that adhered to the grammar, while seeing a grid of six non-words presented on a computer screen. Participants were required to simultaneously listen to the speech stream and click on the corresponding non-words in the grid. Results showed that participants were faster to respond to non-words in predictable positions (i.e., element *b* in the *a*X*b* structure) than in unpredictable positions (i.e., element *a* in the *aXb* structure). Similar to results from the SRT task, this effect of RT on position disappeared in a subsequent trial block where the non-adjacent dependencies present in preceding blocks were violated. These results reflect participants’ sensitivity to the distinction between predictable and unpredictable elements within the speech stream. Note, however, that this method is not suitable for use with early-school-aged children as it requires advanced literacy skills and is not suited for testing SL in the visual modality.

Another proposed online method that uses RTs to investigate the trajectory of auditory SL is asking participants to detect clicks within the speech stream while recording the RTs to this click detection task (Gómez et al., 2011). By presenting clicks both within and between words in the speech stream, Gómez et al. (2011) showed that participants were faster to respond to clicks between words than within words. They argued that these findings are due to participants’ expectations based on the TP structure in the stream (i.e., within words TPs are high and thus participants expect the following syllables, which is not the case between words), thereby reflecting sensitivity to the TP structure.

In 2018, an online target detection task was used in two SL tasks: one in the auditory and one in the visual domain (Qi et al., 2018). Participants were exposed to a stream of stimuli, which were organized into triplets (Saffran et al., 1996). In this TP structure, the occurrence of elements 2 and 3 within triplets is predictable, whereas element 1 within triplets is unpredictable (e.g., in the triplet *ABC*, elements *B* and *C* are predictable after the presentation of *A*, but the first element of the subsequent triplet, e.g., *D* in the triplet *DEF*, is unpredictable since the presentation order varies between triplets). The target task held that participants were required to respond with a button press to 1 out of 12 stimuli presented to them. The target was always the third stimulus in a group of three and was thus a predictable stimulus. The results showed an acceleration of RTs in detecting the targets in the visual, but not auditory modality, in both adult and child (mean age = 12.2) participants, which was taken to reflect learning in the visual task. In this experimental set-up, however, nothing is known about the RTs to non-target trials (i.e., the first and second stimulus in each group of three). It could be the case that a similar acceleration in RTs would appear for these stimuli, which would indicate accommodation to the task in general (i.e., a practice effect) instead of sensitivity to the TP structure during the learning process.

Finally, and most relevant to the present study, two recent studies have applied the self-paced reading method to SL in the visual domain (Karuza et al., 2014; Siegelman et al., 2018). This approach allows participants to control the rate of exposure during familiarization by letting them press a button each time they want to proceed to the following stimulus. In this paradigm, RTs to each individual stimulus are recorded, allowing for the direct comparison of RTs to predictable versus unpredictable stimuli. Karuza et al. (2014) tested adult participants on a visual self-paced non-adjacent artificial grammar learning task containing strings of the form *aXb* and showed that predictable elements yielded shorter RTs than unpredictable elements, thus corroborating the findings by Misyak et al. (2010). Similarly, Siegelman et al. (2018) assessed learning in the visual triplet learning task. In line with previous findings, and following their predictions, results show that adults respond slower to unpredictable stimuli (element 1 within triplets) than predictable stimuli (elements 2 and 3 within triplets). The question of whether a similar RT measure of VSL could be employed in child research is yet unanswered.

In sum, previous studies have shown that online measures are an important tool to study learning during the familiarization phase of SL experiments and provide additional insights into an individual’s performance. In the present study we therefore aim to investigate whether RTs to individual stimuli during familiarization, as introduced by Karuza et al. (2014) and Siegelman et al. (2018), could be used to assess learning in early-school-aged children (perhaps in addition to traditional offline measures). There are several important differences between children and adults that should be taken into account in the assessment of their behavior, one of them being the control of attention. Young children are immature with respect to attentional control compared to adults (Garon et al., 2008). Since attention is a critical component of SL (Baker et al., 2004; Toro et al., 2005; Arciuli, 2017), a secondary aim was to find out whether a cover task that attracts children’s attention to the VSL task (responding to a deviating visual stimulus) influences their learning performance. Although cover tasks have been used in VSL experiments with children and adults to ensure that participants’ attention is targeted to the stimulus stream (e.g., Arciuli and Simpson, 2011), the effect of the presence (or absence) of a cover task on learning performance in VSL tasks has not yet been investigated.

### The Current Study

To test whether online measures of SL are a useful method to investigate learning in child participants, we conducted a study of children’s performance on an SL task containing both online and offline measures. Our main aim was to test whether the online RT measure introduced by Siegelman et al. (2018) is able to assess SL in early-school-aged children by employing a child-adapted version of their self-paced VSL task and could thus be used in addition to more traditional offline measures. The offline test phase consisted of two parts: next the conventional 2-AFC questions, we included 3-AFC questions in which children were required to complete triplets by choosing one out of three possible stimuli (Bertels et al., 2012, 2015; Siegelman et al., 2017b). Our secondary aim was to assess the effect of a cover task on children’s learning in the self-paced visual SL task. Half of the participants completed the self-paced VSL task with cover task (Arciuli and Simpson, 2011), while the other half completed the same experiment without cover task. Therefore, our analyses of the self-paced VSL task were aimed to answer the following research questions:

(1) Can we use the online RT measure of the self-paced VSL task to assess learning in early-school-aged children?
(2) Can we use the offline test performance of the self-paced VSL task to assess learning in early-school-aged children?
(3) Do children who receive a cover task during the self-paced VSL task perform differently on the on- and offline measures of learning than children who do not perform a cover task?

If early-school-aged children are sensitive to the TP structure of the stimulus sequence presented to them in the self-paced VSL task, we expect them to respond more slowly to unpredictable elements (i.e., element 1 of a triplet) than to predictable elements within triplets (i.e., elements 2 and 3), in line with the results obtained with adults (Siegelman et al., 2018). Furthermore, learning in the online measure could be reflected in an interaction between the difference in RT to unpredictable versus predictable elements and the effect of time, since learning is likely to develop during the task. Regarding the second research question, if early-school-aged children are sensitive to the TP structure of the stimulus stream and are able to express this knowledge in an offline testing situation, we expect them to perform above chance-level on these question types (i.e., proportion correct above ^1^/_2_ in 2-AFC questions and above ^1^/_3_ in 3-AFC questions). As for the effect of a cover task on learning outcomes, we hypothesize that the cover task increases the attention paid to the task, thereby having a positive influence on learning. However, since Franco et al. (2015) found that paying attention to deviating stimuli in the form of a click detection task impaired (offline) performance, it could also be the case that performing the cover task is detrimental to learning.

Finally, the relationship between performance on the three measures of learning used in the present study (online RT, offline 2-AFC, and offline 3-AFC) was examined as part of our exploratory analyses. If it is the case that all measures of learning represent the same underlying construct (i.e., children’s sensitivity to the TP structure), we expect to find correlations between all measures. However, we may encounter some difficulties measuring children’s sensitivity to the TP structure in offline measures, as offline performance may rely on alternate processes such as explicit decision making. Therefore, this may result in the absence of a correlation between the online and offline measures. Alternatively, low correlations between on- and offline measures could be the result of differential underlying components of SL (e.g., online measures may reflect implicit learning processes whereas offline measures may tap into more explicit knowledge; see e.g., Bertels et al., 2012, 2015; Siegelman et al., 2017a).

## Materials and Methods

### Participants

Dutch-speaking children were recruited from grades 1 and 2 in four primary schools located in four different provinces of the Netherlands. From the original sample of 54 children, 1 child was excluded due to equipment failure. Thus, the final sample consisted of 53 participants (26 girls and 27 boys) aged between 5;9 and 8;7 (age in years; months, *M* = 7;3, *SD* = 0;6). Fourteen participants attended grade 1, the remaining 29 participants were in grade 2. Twenty-five children (12 girls, 13 boys; mean age = 7;2, *SD* = 0;5) performed the VSL task without cover task, while the other 28 (14 girls, 14 boys; mean age = 7;3, *SD* = 0;7) performed the task with cover task. All participants were native speakers of Dutch, had no hearing problems, and no diagnosis of developmental dyslexia, language impairments, AD(H)D, or autism according to teacher’s reports. The Ethics Committee of the Faculty of Humanities of the University of Amsterdam approved the present study in 2016. Compliant with the regulations of the ethics committee, parents, and/or legal guardians of the children attending grades 1 and 2 in the participating schools were informed about the research project through a newsletter and had the possibility to retract permission of including their child in the study up until 8 days after testing (i.e., passive consent).

### Materials and Design

The VSL task consisted of a familiarization phase and a subsequent offline test phase as is typical for SL tasks. The structure of the current VSL task was similar to that used in several previous studies (e.g., Arciuli and Simpson, 2011, 2012). The task consisted of 12 visual stimuli that could be described as aliens, which were organized into four groups of three (i.e., triplets). These four triplets are referred to as *ABC*, *DEF*, *GHI*, and *JKL* (see the Supplementary Material).

#### Familiarization Phase

**FIGURE 1:**
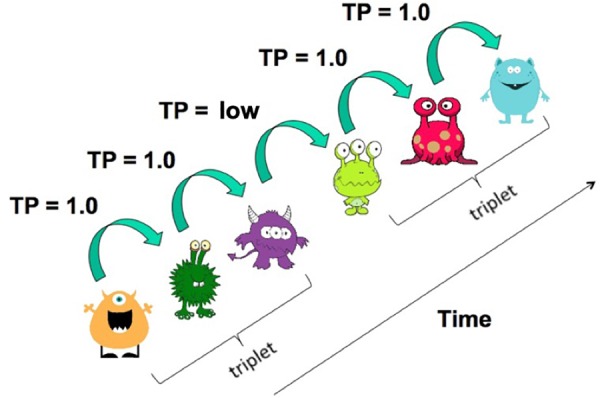
Illustration of the VSL stimuli and the triplet and TP structure.

During familiarization, each alien was presented individually on the screen of a Surface 3 tablet with touch screen. Unbeknownst to the participant, each alien was part of a triplet that always occurred in the same order (i.e., in the triplet *ABC*, *B* always followed *A* and *C* always followed *B*). The four triplets were presented 24 times each, divided into four blocks comprising 6 repetitions per triplet. Four blocks were created so that children could take a short break in between blocks, which aimed to help them stay focused on the task. This resulted in a total of 96 triplets and 288 presentations of individual aliens. Two lists of randomized orders of presentation were created to control for potential effects of order of presentation. This randomization was constrained in two ways: (1) the same triplet was not allowed to appear twice in a row (e.g., *ABC*, *ABC* was forbidden), and (2) pairs of triplets were not repeated (e.g., *ABC*, *JKL*, *ABC*, *JKL* was forbidden) (Turk-Browne et al., 2005; Arciuli and Simpson, 2011). As a consequence, elements 2 and 3 of a triplet are fully predictable (with TP = 1 for one alien and TP = 0 for the remaining 11, henceforth “predictable elements”), whereas element 1 of a triplet is less predictable (TP ≈ 0.4 for three aliens, TP = 0 for the remaining 9, henceforth “unpredictable elements”). Thus, TPs within triplets are high (from element 1 to element 2 and from element 2 to element 3), whereas TPs between triplets are low (from element 3 of triplet *i* to element 1 of triplet *i* + 1). Figure 1 illustrates the TP structure of the VSL task.

Importantly, a novel addition to the present VSL experiment was the use of an online RT measure during the familiarization phase. Following Siegelman et al. (2018), participants determined the speed of presentation of each individual alien themselves by pressing the space bar every time they wanted to proceed to the next stimulus. After each press on the space bar, the following stimulus appeared after 200 ms. Due to time constraints during testing, presentation proceeded to the next stimulus when participants did not respond within 10 s and these trials were not included in the analyses. RTs for each space bar press were recorded for all participants and served as the online measure of SL, which was used to investigate the effects of learning during the familiarization phase. It was hypothesized that, if early-school-aged children are sensitive to the TP structure, RTs to unpredictable elements (i.e., element 1 within triplets) would be slower than RTs to predictable elements (i.e., elements 2 and 3 within triplets; see Siegelman et al., 2018).

In order to investigate whether including a cover task in the VSL influenced participants’ online and/or offline performance, half of the participants received a version of the VSL that included a cover task during the familiarization phase (Arciuli and Simpson, 2011, 2012). In the version of the experiment without cover task, the familiarization phase consisted of the continuous presentation of individual aliens that, unknown to the participant, adhered to the TP structure. In the version of the experiment with cover task, a deviant stimulus (the *intruder alien*) was presented four times per block at random positions in between triplets (i.e., preceding 16.7% of all triplets in a block) and participants were required to press the intruder alien on the touchscreen to proceed. This intruder alien was always the same visual stimulus that was not part of the set of 12 stimuli that were used to form the triplets. Importantly, the deviant stimulus was presented in random positions in the sequence, but only between – and thus not within – triplets. RTs to the triplet following the presentation of the intruder alien were not included in the analysis of the online measure of SL, as these were likely to deviate from the overall RTs.

#### Offline Test Phase

After the familiarization phase, participants were tested on their knowledge of the triplets presented to them (the *base triplets*) in an offline test phase that consisted of 40 multiple-choice questions. Using the aliens of the four base triplets, four new triplets were created that had never appeared during familiarization (the *foil triplets*). These foil triplets did not violate the position of the stimuli in the base triplets (e.g., a stimulus that appeared in the first position in the base triplet, also appeared in the first position of a foil triplet) and are referred to as *AEI*, *DHL*, *GKC*, and *JBF*. Whereas the TPs between aliens within the base triplets were 1, the foil triplets were constructed from pairs of aliens that had a TP of 0 during training. The test phase contained two parts, both containing multiple choice questions: (1) 24 2-AFC trials in which participants were asked to pick the familiar pattern (*pattern recognition* trials, chance level = ^1^/_2_), and (2) 16 3-AFC trials that required the participants to complete a missing stimulus in a pattern (*pattern completion* trials, chance level = ^1^/_3_). Test items either tested complete triplets (pattern recognition: *N* = 8, pattern completion: *N* = 8) or pairs within each triplet (pattern recognition: *N* = 16, pattern completion: *N* = 8) in order to include items that had differing properties and levels of difficulty (see Siegelman et al., 2017b). Each base triplet (e.g., *ABC*) is tested twice: in one trial it is contrasted with a foil triplet that does not contain any of the same elements (e.g., *DHL*) and in one trial with a foil triplet that contains one of the same elements (e.g., *GKC*). The same holds for each pair within base triplets (e.g., *AB* is contrasted with *DH* and *JB*). The frequency of foil triplets, pairs, and single aliens was controlled for (see the Supplementary Material for a complete overview of test items). Additionally, the position of the correct answer on the screen was controlled for and, as in the familiarization phase, two lists of randomized orders of presentation were created. Since foil triplets and pairs occurred equally frequently in the offline test phase as the base triplets and pairs, participants were not able to continue to learn during the 2-AFC questions as the opportunity to learn during testing would be equal for both base and foil triplets (Arciuli and Simpson, 2011, 2012). In all trials, possible answers were presented simultaneously on the screen and participants were instructed to choose the answer that was correct by pressing the screen. Instructions and a practice item preceded both test phases. During the instructions and practice items, participants were encouraged to guess in case they were not certain of the correct answer.

#### Exit Questionnaire

Following the offline test phase, half of the participants completed an exit questionnaire aimed at gaining insight into their explicit awareness of the TP structure. Consequently, information concerning explicit awareness of the VSL is available for half of the participants. The remainder of the participants completed a similar questionnaire about a NADL task, the results of which are described elsewhere as this task was not tested as part of the research questions of the present study (see the section “Procedure” on the procedure of the present study and see Lammertink et al. (2019) for a discussion of the NADL results).

While some of the questions probed the strategies participants used, others directly asked whether participants had any explicit knowledge of the TP structure. For example, questions asked what participants were focused on during familiarization (i.e., were they focusing on the order? Or were they focused on catching the intruder in the case of receiving the version of the experiment with the cover task?), and on what strategy they applied during the test phase (e.g., did they know the answers or were they guessing?). Questions aimed at explicit knowledge of the TP structure included the question whether children noticed that the aliens stood together in groups and whether they could indicate how many aliens stood together in these groups.

### Procedure

Each participant performed three tasks: the VSL task, a spelling test, and an auditory NADL task. As mentioned, the latter tasks were not tested as part of the research questions of this article and are therefore not presented here (but see Lammertink et al., 2019).

The order of the tasks was controlled: half of the participants performed the VSL before the NADL and the other half vice versa. Additionally, half of the participants that received the VSL as their first task performed the version with the cover task and the other half completed the version without the cover task. The same holds for those participants that received the VSL as the last task. Finally, two random orders of appearance were created to which participants were randomly assigned. The spelling task was always administered between the VSL and NADL tasks. In total, this resulted in a list of eight orders to which participants were randomly assigned. As mentioned in the section “Exit Questionnaire,” once participants had completed all tasks, they were asked several questions probing their explicit awareness of the structure of the last task (VSL or NADL) they performed.

Prior to the familiarization phase of the self-paced VSL, participants were informed that they would see aliens standing in line one at a time and that they were waiting to go home in a space ship. They were instructed to send each alien home by pressing the space bar and were informed that the next alien standing in line would appear automatically. Importantly, they were told that some of the aliens really like each other and would stand in line together. Participants were instructed to watch each alien closely and to pay attention to the order of the aliens, because they would receive questions about this later (these instructions were in line with those provided in studies with child participants by Siegelman et al., personal communication). Following these instructions, participants would practice the task during a practice phase containing 12 randomly ordered aliens in order to familiarize them with the procedure. The aliens included in the practice phase were different stimuli than those used in the familiarization phase. In the version of the VSL with cover task, participants received additional instructions regarding the intruder alien. The intruder alien was depicted on the screen and participants were told that this was an intruder alien that was not allowed on the spaceship. When participants saw this intruder alien, they would have to scare it away by touching it on the screen. This was followed by an additional practice round of 12 randomly ordered aliens and 3 randomly placed intruder aliens, during which participants were instructed to pay attention to the order of the aliens and to scare away the intruder aliens. Before completing the offline test phase, children were reminded of the fact that some aliens liked each other and stood in line together and were told they would receive some questions about this. An overview of the original Dutch instructions, with English glosses, is given in the Supplementary Material.

The VSL task lasted approximately 10 min in total, depending on participants’ RTs to the aliens in the familiarization phase and the subsequent multiple-choice questions. In between blocks of the familiarization phase, participants had a break in which they could choose a sticker for a diploma. In the version of the task with the cover task, feedback was given on the number of times the participant caught the intruder alien. The exit questionnaire lasted approximately 3 min.

Children were individually tested in a quiet room at their school in a test session that lasted approximately 60 min. Each participant received stickers on a diploma as a reward for their participation. The VSL task was programmed and ran using E-prime 2.0 software (Psychology Software Tools Inc., 2012; Schneider et al., 2012) on a Surface 3 tablet with touchscreen and keyboard. Instructions were recorded by a female native speaker of Dutch and played over headphones (Sennheiser HD 201).

### Scoring and Analysis

For more detail on our on- and offline analyses and the model outcomes, you can access the raw data, *R* Markdown and/or HTML files through the following link to our project page on the Open Science Framework: https://osf.io/ej32s/.

#### Online Reaction Time Data

Prior to analysis, unreliable measurements were removed from the raw RT data. As mentioned, RTs to the triplet following the appearance of the intruder alien in the cover task were removed, as these RTs are likely to deviate from the other responses (16.7% of the data for children who performed the task with detection cover task). For similar reasons, responses to the first triplet of each of the four blocks of the experiment were excluded from analysis (4.2% of data). Finally, responses faster than 50 ms were removed from the dataset as these reflect cases in which the participant pressed the space bar without processing the stimulus (2.1% of data; element 1: *N* = 89, element 2: *N* = 106, element 3: *N* = 86).

Following pre-processing of raw RTs, the online RT data were analyzed using linear mixed effect models by applying the *lme4* package (Version 1.1-13; Bates et al., 2015) for *R* software (R core Team, 2016). The dependent variable was the RT to each individual alien and was fitted as a function of the within-participant predictors Element (element 1, element 2, and element 3 within triplets) and Time (repetitions 1–24 of the triplets, which was centered and scaled), and Cover (yes or no cover task) as the between-participants predictor. Since the age of children varied between 5;9 and 8;7, age (centered and scaled) was entered as an exploratory between-participants predictor. The two random orders of the task were also entered into the model to take away any variance associated with this contrast (Random Order 1 and Random Order 2). The model contained the maximal random effect structure that did not result in (near-)perfect correlations between the random effects (see Barr et al., 2013) and contained by-subject and by-item^1^ random intercepts and by-subject random slopes for Element and Time^2^ and by-item random slopes for Cover. Age was not entered as by-subject random slopes, since this predictor naturally correlates perfectly with the by-subject intercepts. Note that the *lme4* package provides *t*-values for linear mixed effect models. Confidence intervals (CIs) and the associated *p*-values were calculated through the “profile” function (*lme4* package) and a “get.p.value” formula created for this purpose (see Open Science Framework).

#### Offline Accuracy Data

Responses on the offline test phase were coded as 1 (correct) or 0 (incorrect) for both the 2-AFC pattern recognition questions (maximum score = 24 correct) and the 3-AFC pattern completion questions (maximum score = 16 correct). Results are presented as the proportion of questions answered correctly, ranging from 0 to 1, such that chance level for the 2-AFC questions is ^1^/_2_ and for the 3-AFC questions is ^1^/_3_. None of the responses in the offline test phase were removed from analysis.

Offline accuracy data were analyzed using generalized linear mixed effects models for the 2-AFC and 3-AFC questions separately. The dependent variable was the accuracy of each test item (coded as 1 or 0) and was fitted as a function of Cover (yes or no cover task), Random Order (1 or 2), and Age (centered and scaled) as the between-participants predictors. The models contained by-subject random intercepts. The effect of cover task or age is interpreted as significant if the CI of the log odds does not contain zero.

#### Relationship Between On- and Offline Measures

In order to investigate the relationships between the three measures used in the present study, we ran exploratory correlational analyses using the “cor.test” function with *Pearson* method in R. For the online RT measure, an individual measure of learning was calculated for each participant such that response times to predictable elements were subtracted from RTs to unpredictable elements [RT Element 1 – (RT Element 2 + RT Element 3/2); see Siegelman et al., 2018]. Positive individual RT difference scores thus indicate sensitivity to the TP structure, as these indicate faster responses to predictable than to unpredictable elements. For the offline measures, raw accuracy scores on the 2-AFC and 3-AFC questions were used in correlational analyses.

## Results

We will first focus on the online RT measure in the section “Online Reaction Time Data,” followed by the results of the offline accuracy in the section “Offline Accuracy Data.” Sections “Online Reaction Time Data” and “Offline Accuracy Data” will present confirmatory results, which answer our research questions, and subsequently address several exploratory results obtained through our linear mixed-effects analysis. Additional exploratory analyses, including investigations of correlations between the different measures and inspections of their stability, are presented in the section “Exploratory Results: Relationship Between On- and Offline Measures.” The exploratory results describe either unexpected findings or findings for which no prior hypotheses were constructed (cf. Wagenmakers et al., 2012). The results regarding the exit questionnaire are of a purely descriptive nature and are presented in the section “Exit Questionnaire.”

Importantly, as we used multiple measures in assessing our research questions, all CIs aimed at answering our research questions were Bonferroni-corrected for multiple testing. Thus, CIs were separately adjusted for effects pertaining to evidence of online learning (research question 1), offline learning (research question 2), and the effect of the presence or absence of the cover task (research question 3). To keep the overall false detection rate at 0.05, statistical significance for confirmatory effects regarding research question 1 was determined using 97.5% CIs (i.e., the CI corresponding to a false detection rate of 0.05/2 = 0.025), since two outcomes could provide evidence regarding online learning (i.e., the difference in RTs between predictable and unpredictable elements and this difference in RTs in interaction with Time). Similarly, 97.5% CIs were used for research question 2, since two distinct offline measures were used in the present study (2-AFC and 3-AFC questions). Finally, significance regarding research question 3 was determined using 98.75% CIs (i.e., the CI corresponding to a false detection rate of 0.05/4 = 0.0125), since all four measures could provide an answer regarding the effect of a cover task on learning. For exploratory results we report 95% CIs.

Supplementary analyses were run including the order of the tasks (VSL or NADL first) as a predictor in our models, as requested by an reviewer (see OSF for files containing the supplementary analyses). Task order was found not to interact with the on- and offline measures of learning (all *t-* and *z-*values <1.8). Therefore, we collapse the results from the two testing orders in our presentation of the results.

### Online Reaction Time Data

#### Online Reaction Time Data: Confirmatory Results

In order to answer the first research question of whether children are sensitive to the TP structure present during familiarization, we ran the linear mixed effect model as explained in the section “Online Reaction Time Data.” The effect that is crucial to answering this research question is whether participants responded differently to unpredictable elements (Element 1) than predictable elements (Elements 2 and 3) within triplets. Thus, the three levels of the within-participant predictor Element were coded into orthogonal contrasts such that the first contrast (“Element 1 vs. Elements 2 and 3,” with Element 1 coded as −2/3 and Elements 2 and 3 coded as +1/3) estimated how much the RTs to predictable element 1 within triplets across the task differ from the mean RTs to unpredictable elements 2 and 3, which will allow us to answer our research question. The second contrast of the predictor Element estimated how much the RTs to element 2 differed from the RTs to element 3 (i.e., the two unpredictable elements, with Element 2 coded as −1/2 and Element 3 coded as +1/2), the results of which are described under the section explaining our exploratory findings. The secondary effect that could answer our first research question is the interaction between the difference in RT to predictable versus unpredictable elements and Time (i.e., repetitions of triplets in the experiment), as an increase in the difference between predictable and unpredictable elements by time would indicate increasing responsivity to the TP structure across the experiment. Our third research question regarding the effect of the cover task was tested through interactions between the effect of the orthogonally contrast-coded predictor Cover (with no cover coded as −1/2 and cover coded as +1/2) and the abovementioned effects of learning (i.e., the two-way interaction between Cover and the contrast “Element 1 vs. Elements 2 and 3” or the three-way interaction with the contrasts “Element 1 vs. Elements 2 and 3” and Time).

The model was first run on raw RTs, but the resulting model’s residuals were non-normally distributed. Thus, we attempted using log-transformed RTs and normalized RTs to improve the data’s suitability for analysis using linear mixed effects models. Normalization was performed by sorting all *N* observations in increasing order, then replacing each observation by the (*r* – 0.5)/*N* quantile of the normal distribution, where *r* is the ranking number of the observation; we consequently obtain values that can be interpreted as optimally distributed *z*-values. Through inspections of QQ-plots of the model’s residuals, it was decided that normalized RT data resulted in the best approximation of normally distributed residuals (for more detail: see the R markdown and/or HTML file containing all analyses on the Open Science Framework). For this reason, analyses were run on normalized RT data and the model estimates are expressed as changes in *z*-values (Δ*z*) from one level of the predictor to the next.

**FIGURE 2:**
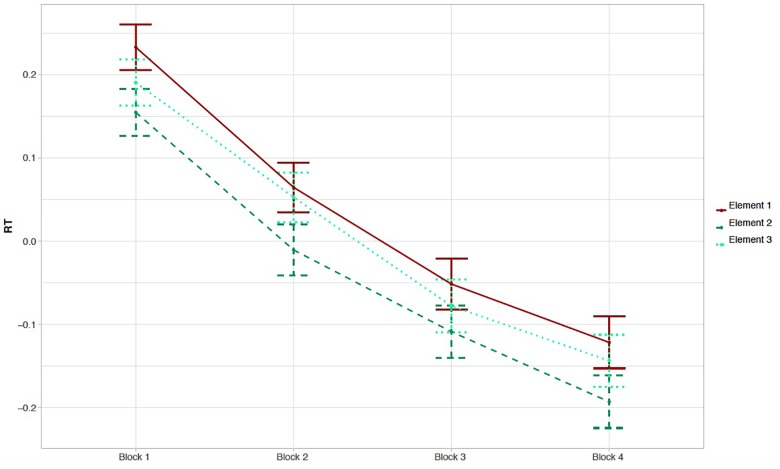
Descriptive results of the online RT data: blocks. Mean normalized RT (±1 SE) to element 1 (unpredictable), element 2, and element 3 (predictable elements) are plotted per block of the experiment.

**FIGURE 3:**
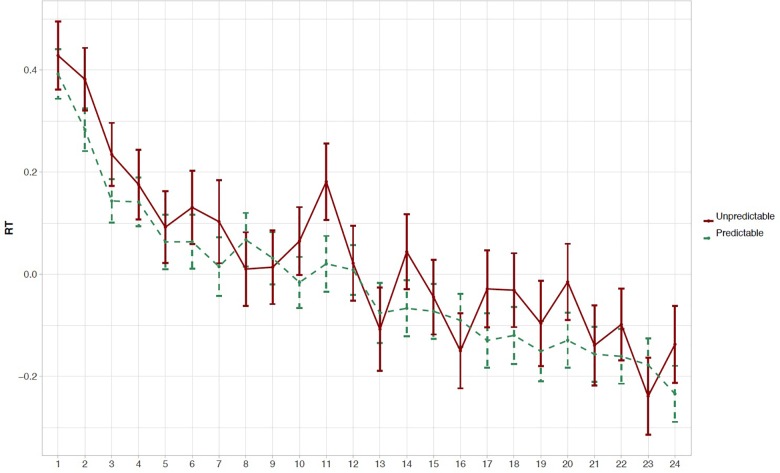
Descriptive results of the online RT data: repetitions. Mean normalized RT (±1 SE) to unpredictable elements (element 1) and predictable elements (average of elements 2 and 3) are plotted per repetition of triplets during the experiment.

**FIGURE 4:**
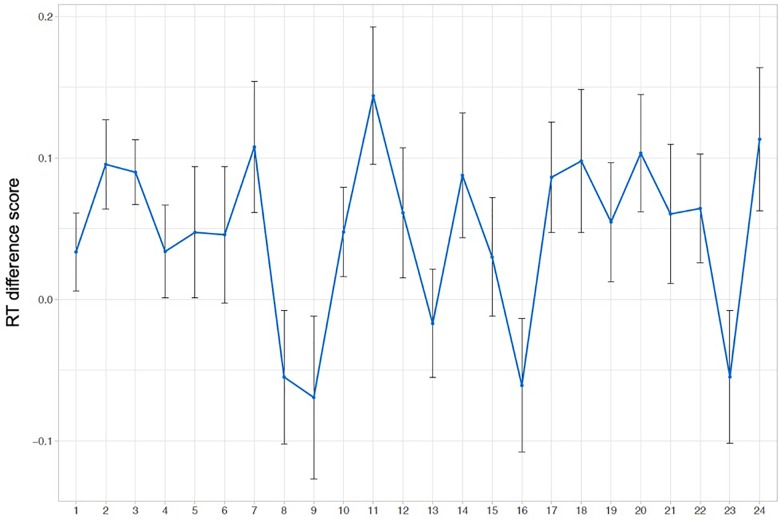
Descriptive results of the online RT data: difference score. Mean normalized RT to unpredictable elements (element 1) minus mean normalized RT to predictable elements (average of elements 2 and 3) plotted per repetition of triplets during the experiment.

Figure 2 presents the normalized RTs to elements 1, 2, and 3 within triplets over the four blocks of the experiment. Note that the normalized RTs in Figure 2 are averaged over blocks, which deviates from the way the analysis was conducted (i.e., on normalized RTs and using a continuous Time predictor as explained in the section “Online Reaction Time Data”). As hypothesized, analysis of normalized RTs reveals that RTs to the unpredictable element 1 within triplets are significantly longer than the mean RT to both predictable elements 2 and 3 [Δ*z* = −0.058, *SE* = 0.022, *t* = −2.605, 97.5% CI = (−0.114 … −0.002), *p* = 0.021], reflecting that early-school-aged children are sensitive to the TP structure presented in the VSL task. The model estimate of the interaction with Time was not significantly different from zero [Δ*z* = −0.004, *SE* = 0.011, *t* = −0.328, 97.5% CI = (−0.028 … +0.021), *p* = 0.74]. An overview of all model estimates is presented in Table 1. The same model was run on raw and log-RT data, resulting in similar *t*-values for the effect of unpredictable element 1 versus both predictable elements 2 and 3 (*t* = −2.074 and *t* = −2.590, respectively). Thus, the reported effect of the predictability of elements within triplets on RTs is stable across models. We did not find evidence for the effect of Element changing over the time course of the task. Figures 3, 4 provide more information regarding the time course of the experiment: Figure 3 plots the normalized RTs for unpredictable (Element 1) and predictable (Elements 2 and 3) stimuli across repetitions of triplets (1–24), while Figure 4 plots the online measure of learning (i.e., difference score: normalized RT Element 1 – mean normalized RT Elements 2 and 3) across repetitions of triplets (based on Figure 3 in Siegelman et al., 2018, p. 702).

**TABLE 1:** Fixed effects of the online normalized RT model, reporting on 13,004 observations by 53 participants across 12 items (i.e., aliens).

	**Estimate (Δ*z*)**	**Standard error (*SE*)**	***t*-value**
(Intercept)	−0.002	0.098	−0.019
**El1 vs. El2 and 3^∗^**	**−0.058**	**0.022**	**−2.605**
*El2 vs. El3^†^*	+*0.053*	*0.026*	+*2.056*
*Time*^∗^	−*0.146*	*0.028*	−*5.184*
Cover	−0.142	0.195	−*0.726*
*Age*^∗^	+*0.269*	*0.105*	+*2.563*
**El1 vs. El2 and 3: Time**	**−0.003**	**0.011**	**−0.328**
El2 vs. El3: Time	+0.002	0.013	+0.125
**El1 vs. El2 and 3: Cover**	**+0.021**	**0.023**	**+0.940**
El2 vs. El3: Cover	−0.014	0.027	−0.508
*El1 vs. El2 and 3: Age*	−*0.012*	*0.012*	−*0.949*
El2 vs. El3: Age	+0.010	0.014	+0.718
**El1 vs. El2 and 3: Time: Cover**	**−0.003**	**0.022**	**−0.156**
El2 vs. El3: Time: Cover	+0.027	0.025	+1.090
*El1 vs. El2 and 3: Time: Age*	+*0.004*	*0.012*	+*0.375*
El2 vs. El3: Time: Age	+0.009	0.013	+0.688

*Model estimates that differ significantly from zero are indicated with an asterisk (^∗^), while estimates that differ marginally significantly from zero are indicated with a cross (^†^). El, element. Effects that were used to answer the research question are marked in bold, while effects explained under exploratory results are marked in italics.*

Our secondary research question pertains to the effect of cover task: do early-school-aged children who receive the self-paced VSL task with a cover task respond differently from children who perform the task without a cover task? Whether the version of the task made a difference in participants’ sensitivity to the TP structure is reflected in the interaction between the between-subjects predictor Cover and the first Element contrast (“Element 1 vs. Elements 2 and 3”). This interaction model estimate did not significantly differ from zero [Δ*z* = 0.021, *SE* = 0.023, *t* = +0.940, 98.75% CI = (−0.036 … +0.079), *p* = 0.35]. Equally, the three-way-interaction with Time also did not differ significantly from zero [Δ*z* = −0.003, *SE* = 0.022, *t* = −0.156, 98.75% CI = (−0.057 … +0.051), *p* = 0.88]. We therefore have no evidence that early-school-aged children perform the online RT task with a cover task differently than the version without a cover task.

#### Online Reaction Time Data: Exploratory Results

Besides allowing us to answer our research questions, the RT model provides some interesting exploratory results that are also evident in the normalized RTs presented in Figures 2–4. Firstly, related to the TP structure of the task, we found that RTs to predictable element 2 within triplets were shorter than RTs to predictable element 3 within triplets, an effect that almost reaches significance [Δ*z* = 0.053, *SE* = 0.026, *t* = +2.056, 95% CI = (−0.002 … +0.108), *p* = 0.058]. If this effect were real, this would mean that the difference between elements 1 and 2 is greater than the difference between elements 1 and 3, which may tell us that children predict element 2 more easily than element 3, although both elements 2 and 3 have a TP of 1 (see the section “Familiarization Phase”). As requested by a reviewer, a additional figure was created plotting the time course of the experiment as in Figure 4 but excluding element 3 (i.e., normalized RT element 1 – normalized RT element 2; see Supplementary Material and our OSF project page).

Secondly, we see that RTs overall, thus ignoring effects of TP structure, significantly decrease as a function of Time [Δ*z* = −0.146, *SE* = 0.028, *t* = −5.184, 95% CI = (−0.203 … −0.090), *p* = 3.11.10^–06^]. This effect of time on RTs is to be expected, as participants respond faster overall as a result of them adapting to the task and needing less time to process each individual stimulus. Finally, regarding the exploratory between-participants predictor Age (ranging between 5.9 and 8.7), the model shows that older children had significantly slower RTs overall [Δ*z* = 0.269, *SE* = 0.105, *t* = 2.563, 95% CI = (+0.131 … +0.481), *p* = 0.0052], likely due to the fact that the older children in our sample have more developed academic skills and are therefore better at focusing on the task at hand. More importantly, however, we find no significant interactions between participants’ age and the difference in RTs to predictable versus unpredictable stimuli or a three-way interaction between age, predictability, and time [Δ*z* = −0.012, *SE* = 0.029, *t* = −0.949, 95% CI = (−0.036 … +0.012), *p* = 0.34, and Δ*z* = 0.004, *SE* = 0.012, *t* = 0.375, 95% CI = (−0.018 … +0.027), *p* = 0.71, respectively].

### Offline Accuracy Data

#### Offline Accuracy Data: Confirmatory Results

**FIGURE 5:**
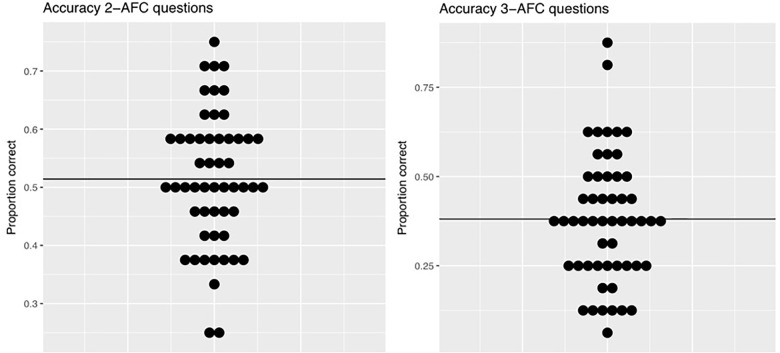
Descriptive results of the offline accuracy data. Distribution of scores for the 2-AFC (left, chance level = ½) and 3-AFC (right, chance level = 1/3) tasks: dots indicate individual mean accuracy scores, black lines represent overall group means.

Following the familiarization phase, participants performed an offline test phase consisting both of pattern recognition (2-AFC, *N* = 24) trials and pattern completion (3-AFC, *N* = 16) trials. Descriptive statistics show that participants scored between 0.250 and 0.750 correct on 2-AFC trials (*M* = 0.514, *SD* = 0.11) and between 0.060 and 0.880 correct on subsequent 3-AFC trials (*M* = 0.381, *SD* = 0.18). Figure 5 shows the descriptive individual and group results on the offline accuracy data for both question types.

The generalized linear mixed effects models were run on the accuracy data as explained in the section “Offline Accuracy Data.” The first research question was whether children can learn the TP structure presented in the VSL task, as measured by their accuracy on the offline test phase. In order to answer this question, we examined whether participants’ accuracy exceeded chance level (i.e., exceeded ^1^/_2_ on 2-AFC and/or ^1^/_3_ on 3-AFC questions). The 2-AFC and 3-AFC model estimated that participants scored 0.015 and 0.037 above chance level, respectively (2-AFC: probability intercept = 0.516, 3-AFC: probability intercept = 0.376). In both cases, this performance was found to not differ significantly from chance, as the correctness probability CIs included the task’s chance probabilities [2-AFC 97.5% CI = (+0.480 … +0.551), *p* = 0.31 and 3-AFC: 97.5% CI = (+0.319 … +0.429), *p* = 0.095]. Hence, we find no evidence of above-chance performance in early-school-aged children on either 2-AFC or 3-AFC questions.

Related to our secondary research question regarding the effect of the cover task, no significant effect of cover task was found on either of the offline measures [2-AFC: odds ratio estimate = 0.927, 98.75% odds CI = (0.673 … 1.274), *p* = 0.54, 3-AFC: odds ratio = 1.140, 98.75% CI = (0.673 … 1.945), *p* = 0.52]. Similar to our findings in the online RT measure, we cannot conclude that early-school-aged children perform the self-paced VSL with a cover task differently than the task without a cover task.

#### Offline Accuracy Data: Exploratory Results

The offline models provide us with exploratory findings regarding the effect of age on performance. No significant effect of age was found on either of the offline measures [2-AFC: odds ratio estimate = +0.097, 95% CI = (−0.037 … +0.233), *p* = 0.15, 3-AFC: odds ratio estimate = +0.131, 95% CI = (−0.103 … +0.373), *p* = 0.27]. Again, in line with our findings in the online model, we find no evidence that age influences the performance of early-school-aged (between 5.9 and 8.7 years of age) children’s performance on the self-paced VSL used in the present study.

### Exploratory Results: Relationship Between On- and Offline Measures

As mentioned in the section “Relationship Between On- and Offline Measures,” we investigated the relationship between on- and offline measures used in the present study. Since we found no effects of time on our online RT measure, the individual RT measure of learning was calculated using the normalized RTs to all stimuli presented during the experiment [normalized RT Element 1 – (normalized RT Element 2 + normalized RT Element 3/2)].

The results show that the two offline accuracy measures correlate significantly with one another [*r* = 0.274, *t*(51) = 2.031, *p*-value = 0.048], and neither of the offline accuracy measures correlate significantly with the online RT measure [2-AFC: *r* = 0.188, *t*(51) = 1.367, *p*-value = 0.178; 3-AFC: *r* = 0.157, *t*(51) = 1.139, *p*-value = 0.26].

### Exit Questionnaire

Subsequent to the offline test phase, half of the participants received a short exit questionnaire (*N* = 24, mean age = 7;4). During familiarization, most children reported paying attention to the aliens’ features (e.g., the color or the number of eyes, *N* = 11) or to the intruder when performing the VSL with cover task (*N* = 6). Five children did not give a clear answer, while the final two claimed to have paid attention to the order in which the aliens appeared. When asked whether children noticed that the aliens continuously appeared in the same groups, the majority of participants responded “no” (*N* = 14), whereas five participants said they did notice the order but could not explain any of the groups when shown pictures of the aliens. Only one participant could recall a single correct triplet and the four remaining children recalled incorrect (or foil) triplets. Most children said they had to guess the answers (*N* = 11) during the offline test phase, while others reported having memorized the correct answers (*N* = 4), or “just knowing” them (*N* = 7). The remaining two children were unable to answer this question. Finally, a large number of children thought groups of aliens consisted of either two or three aliens (*N* = 11), which reflects the use of both pairs and triplet items in the offline test phase. The other 13 children either reported all groups consisted of two (*N* = 3), three (*N* = 6), or four (*N* = 1) aliens, two to four aliens (*N* = 1) or had no idea (*N* = 2). To summarize, the exit questionnaire did not provide evidence of explicit strategies during familiarization or of explicit, verbalizable knowledge of the TP structure as a result of the experiment as a whole.

## Discussion

In the present study, we aimed to test whether a self-paced VSL task using an online RT measure (in addition to traditional offline questions) is a useful method to investigate SL in early-school-aged children. Previous work by Siegelman et al. (2018) has shown the suitability of such a measure for adults, but no study to date has replicated their findings with child participants. In accordance with our hypothesis, results revealed that children between 5;9 and 8;7 years old were sensitive to the TP structure during familiarization as reflected by slower RTs to unpredictable (element 1) versus predictable elements (elements 2 and 3) within triplets. We did not find evidence of an influence of the time course of the experiment on this sensitivity to predictable versus unpredictable stimuli. The reported effect of predictability is in line with previous studies with adult participants showing faster responses to predictable than unpredictable elements in SL tasks, argued to reflect a difference in processing speed between predictable and unpredictable stimuli (Misyak et al., 2010; Karuza et al., 2014; Siegelman et al., 2018). The lack of an interaction with time is supported by other studies reporting that learning takes place early on during exposure (e.g., Hedenius et al., 2013). Similarly, in their investigation of the self-paced VSL with adults, Siegelman et al. (2018) reported significant learning as early as after seven repetitions of triplets. Importantly, this study demonstrates that early-school-aged children show similar sensitivity to predictability during exposure to an SL task. Additionally, the online measure provides information that goes beyond the traditional offline 2-AFC (and 3-AFC) questions, for which we did not find evidence of above chance-level performance. So, while the offline accuracy data do not provide conclusive evidence for sensitivity to TP structure in early-school-aged children, the online RT measure does. This finding highlights the importance of using online measures (possibly in addition to offline measures) when investigating SL in children. Moreover, the fact that the online RT measure of the self-paced VSL task has now been shown to be sensitive to children’s learning abilities allows future studies to compare performance across development using the same task.

The data presented here could not determine whether 5–8-year-old children exceed chance level on the 2-AFC questions. We cannot reject the possibility that the failure of the 2-AFC (and 3-AFC) task could simply be due to chance (the design does not make it possible to directly compare the sensitivities of the three tasks). However, the failure could also be due to low sensitivity of the task when used with young children, which leads to difficulties in reliably measuring learning using the 2-AFC task in this population. Since the CI of the learning effect on the 2-AFC task ranged from 0.480 to 0.551, and the upper bound is thus only a performance of 0.551, we can cautiously conclude that if a learning effect on 2-AFC questions exists in early-school-aged children, it is a very small effect. Additionally, we found no improvement with age in this younger age group. These difficulties with assessing the VSL abilities of young children through the 2-AFC task have been reported before in the literature. In studies that employ a similar VSL task structure as presented here, significantly above-chance learning has been reported in children (Arciuli and Simpson, 2011, 2012). However, whereas children in Arciuli and Simpson (2011) were aged between 5;6 and 12;6 (*M* = 9;5), and between 5;10 and 12;5 (*M* = 9;1) in their 2012 study, children in our study were tested within the lower spectrum of their age ranges (i.e., between 5;9 and 8;7, *M* = 7;3). In their investigations of the effect of participant- and task-related variables on learning performance in a multiple linear regression analysis, Arciuli and Simpson (2011) found that VSL abilities develop between ages 5 and 12: learning performance on the 2-AFC task increased with age. These findings have been replicated in two other samples of children between 5 and 12 years of age, revealing higher mean performance on 2-AFC questions of a VSL task as a function of age (Raviv and Arnon, 2017; Shufaniya and Arnon, 2018). Although these findings may be interpreted as development of VSL *abilities* in these age groups, they may in fact reflect the difficulties of *measuring* children’s abilities using offline measures (or, alternatively stated, they may reflect the development of the ability to make judgments involved in offline measures). This is what our results suggest, since we find evidence of sensitivity to the VSL structure in our online RT measure but no evidence of learning in our offline measures. Our results therefore underline the difficulties in using offline questions with early-school-aged children and underline the importance of using different measures in children, especially in younger age groups, to tap into their sensitivity to structure in SL tasks. Early-school-aged children, as opposed to adults (and infants), may be more likely to develop incorrect strategies when answering offline questions (e.g., focusing on the visual features of the stimuli, as we saw from the exit questionnaire) and are likely more susceptible to distractions during a complex task such as answering 2- and 3-AFC questions. Future research investigating (the development of) VSL in children could apply the online RT measure of learning as proposed here (in addition to offline measures) to obtain a more complete picture of children’s SL abilities.

The secondary aim of this study was to assess the effect of a cover task on children’s performance in the self-paced VSL. Although we hypothesized that the inclusion of a cover task should attract children’s attention to the task, thereby enhancing performance, we did not find any evidence of a positive effect of including a cover task on the offline or online performance of children. Additionally, whereas Franco et al.’s (2015) study reported that paying attention to a deviating stimulus during familiarization impaired adult participants’ offline performance, we do not find evidence for a detrimental effect of our cover task on children’s VSL performance either. Based on our findings, we cannot conclude whether early-school-aged children are affected by the presence or absence of the cover task in a VSL task as the one reported on here. Note that, although the cover task was designed to ensure children’s attention to the VSL task (see also Arciuli and Simpson, 2011), it may be the case that it did not affect children’s attention overall and therefore no evidence of an effect on VSL performance was found. Future studies that aim to investigate the potential effect of a cover task on VSL performance should include an independent measure of attention payed to the task overall to control for this possibility.

Finally, we explored the relationships between the on- and offline measures of learning used in the present study, revealing a relationship between children’s performance on the two distinct offline question types as expected. We found no evidence of a relationship between the online RT measure of learning and offline performance on either 2-AFC or 3-AFC questions. This lack of correlation between online and offline SL measures has been reported before (e.g., Misyak et al., 2010; Franco et al., 2015) and has several possible explanations. Firstly, although both online and offline measures are assumed to measure SL in general, they may tap into different stages or different aspects of the learning process. Whereas online measures assess participants’ (implicit) sensitivity to the TP structure as it is presented to them, offline measures evaluate participants’ ability to make explicit judgments about stimuli subsequent to exposure (e.g., Franco et al., 2015; Siegelman et al., 2018). Therefore, performance on these two separable processes may not be related to one another. As mentioned by Misyak et al. (2010), the online measure is a more implicit and indirect measure of learning, while the offline measure is more explicit and direct. The two types of measures may therefore be “functionally dissociable” (Willingham et al., 1989; Cohen et al., 1990; Destrebecqz and Cleeremans, 2001). This lack of correlation makes even more sense in the current context of early-school-aged children, since young children are known to have difficulties with explicit decision making (Bialystok, 1986). This may have resulted in the lack of evidence of above-chance performance observed in the present study, which in turn may hinder the investigation of the relationship between the different measures of learning in the self-paced VSL task. Offline measures that are more sensitive to the learning outcome of young children need to be developed in order to further explore these relationships in child participants. For example, more indirect and implicit offline measures as developed by Bertels et al. (2012, 2015) may be suitable for future research with early-school-aged children.

Although the current results regarding the online measure of learning in the self-paced VSL are very promising, we see some room for improvement. Importantly, the observed effect of predictability on children’s response times was small and the difference in response times to predictable and unpredictable stimuli varied greatly between individuals. Moreover, we found no evidence of learning developing over time (i.e., an interaction between the measure of learning and the time course of the experiment, expressed as repetitions of triplets). Such an effect of time on learning would be expected theoretically, since it is assumed that participants become increasingly sensitive to the statistical structure as exposure enfolds (e.g., Batterink and Paller, 2017; Siegelman et al., 2018). While the online RT measure appears suitable for group analyses as presented in the current study, the methodology may need to be improved on in order to apply it in an individual differences approach or to investigate the time course of learning in more detail. As suggested by Siegelman et al. (2018), the presented behavioral methods may be used in combination with neurobiological methods such as EEG in order to gain more insight into the online learning process of individuals. Furthermore, methodological changes to the current design may improve the sensitivity of measuring learning online and may allow for closer inspections of the time course of learning. For example, the lack of an interaction between learning and time in the present study may be the result of the introductions of blocks in the experiment or of participants’ lack of attention to the task toward the end. While these blocks were introduced in order to keep children’s attention and motivation to the task, they may have hampered the measurement of the online time course of learning by interrupting the continuous learning process. Additionally, children might need further encouragement to continuously pay attention to the stream of stimuli in this type of SL tasks.

Recently, attention has been paid to the nature of the learning mechanisms underlying performance on SL tasks (e.g., Siegelman et al., 2019). Learning in tasks such as the VSL presented here could be the result of sensitivity to local TPs (i.e., between pairs of stimuli) or may alternatively follow from sensitivity to more global TP patterns [i.e., “chunks” or triplets; see Siegelman et al. (2019) for a discussion]. In their study of adult participants, Siegelman et al. (2019) showed that participants apply both types of learning, and the reliance on one or the other differs across participants. As can be gleaned from Figure 2 and the *p*-value of 0.058 reported in the section “Online Reaction Time Data: Exploratory Results,” the results from the present study may suggest a larger difference between element 1 compared to element 2 than compared to element 3 within triplets, which may be indicative of larger sensitivity to local than to global TPs (i.e., pairs versus triplets) in child participants. Please note that this is highly speculative, since the present study was not set up to differentiate between these two learning mechanisms. However, this line of research opens up avenues for further investigations of the interplay between differing learning mechanisms, both in adult and in child participants. Moreover, the online RT measure of learning is a tool that is potentially useful in such explorations (see also Siegelman et al., 2019).

In sum, the present study underlines the importance of developing novel sensitive measures of SL appropriate for child research and looking beyond traditional offline questions when investigating SL in (early-school-aged) children. Online measures cannot only reveal sensitivity to statistical regularities during familiarization that offline questions cannot, but also have the potential to inform us about the learning trajectories of participants in different SL tasks, although further research is needed to reach this goal. The RT measure of learning presented here provides an implicit, online measure that can detect sensitivity to TP structure during exposure. The self-paced VSL has thus been shown to be a useful tool in assessing learning in children and could be further developed and adapted for future studies investigating developmental patterns of VSL or for use in clinical populations (perhaps besides more traditional offline measures). For example, a number of studies have shown impairments in the area of SL in individuals with developmental language disorders and dyslexia (see e.g., Evans et al., 2009; Gabay et al., 2015). Online measures could provide further information regarding the differences in performance between such populations and their neurotypical peers. Future research could investigate the use of the self-paced VSL for an individual differences approach by exploring the relationship between the online sensitivity to TP structure of individual participants and their performance on language measures.

## Ethics Statement

This study was carried out in accordance with the recommendations of the Ethics Committee of the Faculty of Humanities of The University of Amsterdam. Written informed consent from the participants’ legal guardian/next of kin was not required to participate in this study in accordance with the national legislation and the institutional requirements at the time the study was conducted.

## Author Contributions

MvW, IL, PB, FW, and JR collaboratively designed the study. MvW and IL recruited the participants and collected the child data. MvW and PB analyzed the data. MvW is the lead author of this manuscript, with contributions from PB, FW, and JR.

## Conflict of Interest Statement

The authors declare that the research was conducted in the absence of any commercial or financial relationships that could be construed as a potential conflict of interest.

## Footnotes

1 Item in the online model refers to the individual aliens used in the experiment (*N* = 12).

2 The by-subject random slopes for the interaction between Element and Time were removed from the model, as these random slopes correlated perfectly with the by-subject intercepts indicating that the model was overparameterized. Removing these random slopes was licensed, since the interaction between Element and Time was not significant. Removal did not decrease the fit of the model (χ^2^ = 1.333, *df* = 11, *p* = 0.9998) or change its main outcomes.
