# Supplementary material for: Assessing Visual Statistical Learning in Early-School-Aged Children: The Usefulness of an Online Reaction Time Measure
Source: Front Psychol. 2019 Sep 13;10:2051. doi: 10.3389/fpsyg.2019.02051 (PMC6753232; doi:10.3389/fpsyg.2019.02051)
Supplement: Supplementary file 1 [file Table_1.docx]

Supplementary Material

# Supplementary Material: VSL stimuli

Triplet ABC


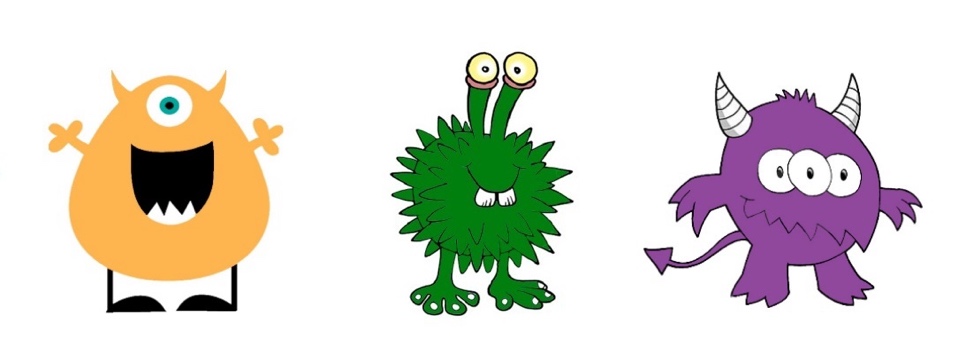


Triplet DEF


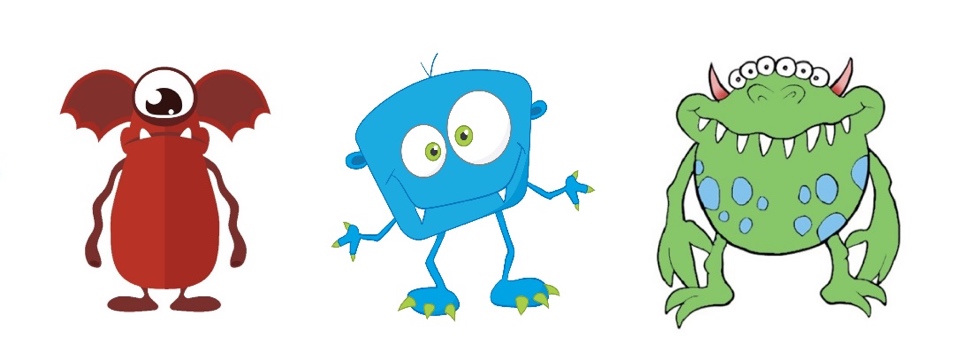


Triplet GHI


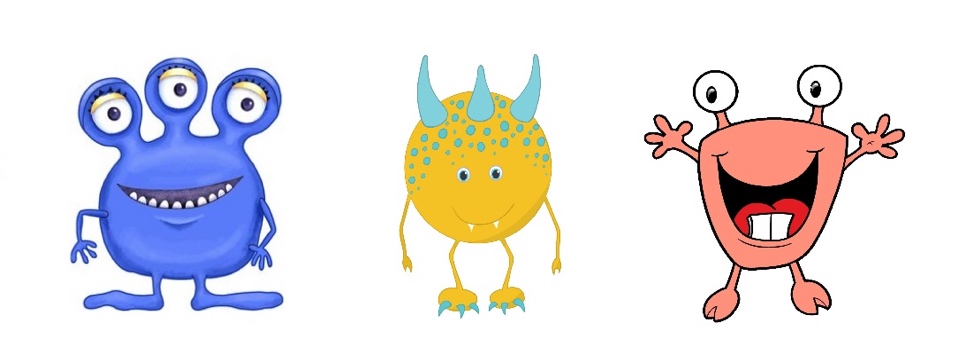


Triplet JKL


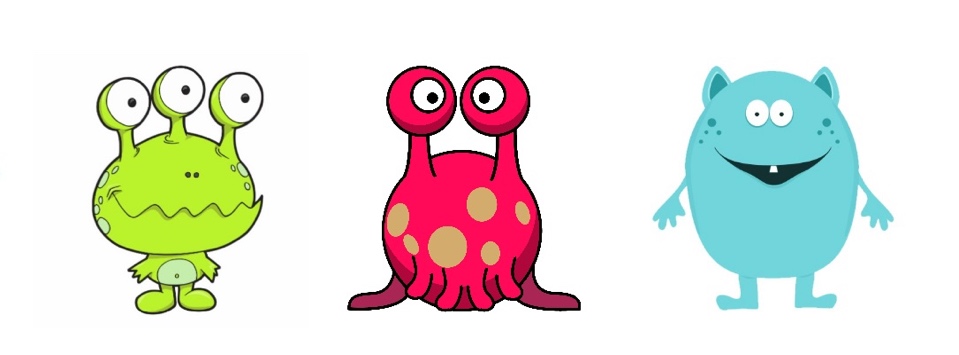


# Supplementary Material: VSL test items

| 2-AFC: pattern recognition | | | | |
| --- | --- | --- | --- | --- |
| **Item** | **Grammatical** | **Ungrammatical** | **Chance** | **Similarity distractor** |
| 1 | **ABC** | DHL | 50% | No |
| 2 | **ABC** | GKC | 50% | Yes |
| 3 | **DEF** | GKC | 50% | No |
| 4 | **DEF** | JBF | 50% | Yes |
| 5 | **GHI** | JBF | 50% | No |
| 6 | **GHI** | AEI | 50% | Yes |
| 7 | **JKL** | AEI | 50% | No |
| 8 | **JKL** | DHL | 50% | Yes |
| 9 | **AB** | JB | 50% | Yes |
| 10 | **AB** | DH | 50% | No |
| 11 | **BC** | BF | 50% | Yes |
| 12 | **BC** | EI | 50% | No |
| 13 | **DE** | AE | 50% | Yes |
| 14 | **DE** | KC | 50% | No |
| 15 | **EF** | BF | 50% | Yes |
| 16 | **EF** | GK | 50% | No |
| 17 | **GH** | DH | 50% | Yes |
| 18 | **GH** | EI | 50% | No |
| 19 | **HI** | HL | 50% | Yes |
| 20 | **HI** | GK | 50% | No |
| 21 | **JK** | JB | 50% | Yes |
| 22 | **JK** | HL | 50% | No |
| 23 | **KL** | KC | 50% | Yes |
| 24 | **KL** | AE | 50% | No |
| 3-AFC: pattern completion | | | | |
| **Item** | **Pattern** | **Answer options** | **Chance** | **Position question mark** |
| 25 | A ? C | **B**, J, H | 33% | 2 |
| 26 | D E ? | **F**, B, C | 33% | 3 |
| 27 | ? H I | **G**, L, A | 33% | 1 |
| 28 | J ? L | **K**, F, E | 33% | 2 |
| 29 | ? B C | **A**, H, G | 33% | 1 |
| 30 | D ? F | **E**, G, B | 33% | 2 |
| 31 | G H ? | **I**, D, L | 33% | 3 |
| 32 | ? K L | **J**, C, E | 33% | 1 |
| 33 | B ? | **C**, E, F | 33% | 1 |
| 34 | ? C | **B**, D, K | 33% | 2 |
| 35 | ? E | **D**, K, J | 33% | 1 |
| 36 | E ? | **F**, G, C | 33% | 3 |
| 37 | G ? | **H**, A, K | 33% | 2 |
| 38 | H ? | **I**, D, L | 33% | 3 |
| 39 | ? K | **J**, I, A | 33% | 1 |
| 40 | K ? | **L**, H, I | 33% | 3 |

# Supplementary Material: VSL instructions

General instructions

*Dutch original: Je ziet straks alle aliens die in de rij staan. Je ziet steeds één alien tegelijk. Stuur de alien naar huis door op de spatiebalk te drukken. Daarna zie je vanzelf de volgende alien in de rij.*

English translation: You will see all of the aliens standing in the line. You will see one alien at a time. Send the alien home by pressing the space bar. Afterwards, you will automatically see the next alien standing in the line.

*Dutch original: In dit spel vinden sommige aliens elkaar heel leuk. Zij staan bij elkaar in de rij. Bekijk elke alien goed en let goed op de volgorde van de aliens, want daarover stel ik je later nog wat vragen.*

English translation: In this game, some aliens really like each other. They stand together in line. Watch each alien closely and pay attention to the order of the aliens, because I will ask you some questions about this later on.

Cover task instructions

*Dutch original: Dit is een indringer! De indringer mag niet mee op het ruimteschip. Als je deze indringer ziet, moet je hem wegjagen. Dit doe je door op hem te drukken. Je kan gewoon met je vinger op het scherm drukken. Probeer maar!*

English translation: This is an intruder! The intruder is not allowed to join the others on the spaceship. If you see this intruder, you have to scare him away. You can do this by touching him on the screen with your finger. Try it!

*Dutch original: Goed zo! Als je de indringer weggejaagd hebt, gaat het spel verder.*

English translation: Well done! When you scare away the intruder, the game continues.

Test phase instructions

*Dutch original: Nu gaan we nog iets anders doen. Sommige aliens vonden elkaar heel leuk en stonden daarom bij elkaar in de rij. Als het goed is, heb jij hierop gelet! Daar krijg je nu een paar vragen over.*

English translation: Now we’re going to do something different. Some aliens really liked each other and stood in line together. Did you pay attention to this? You will now receive some questions about this.

# Supplementary Material: Additional figure as requested by reviewer


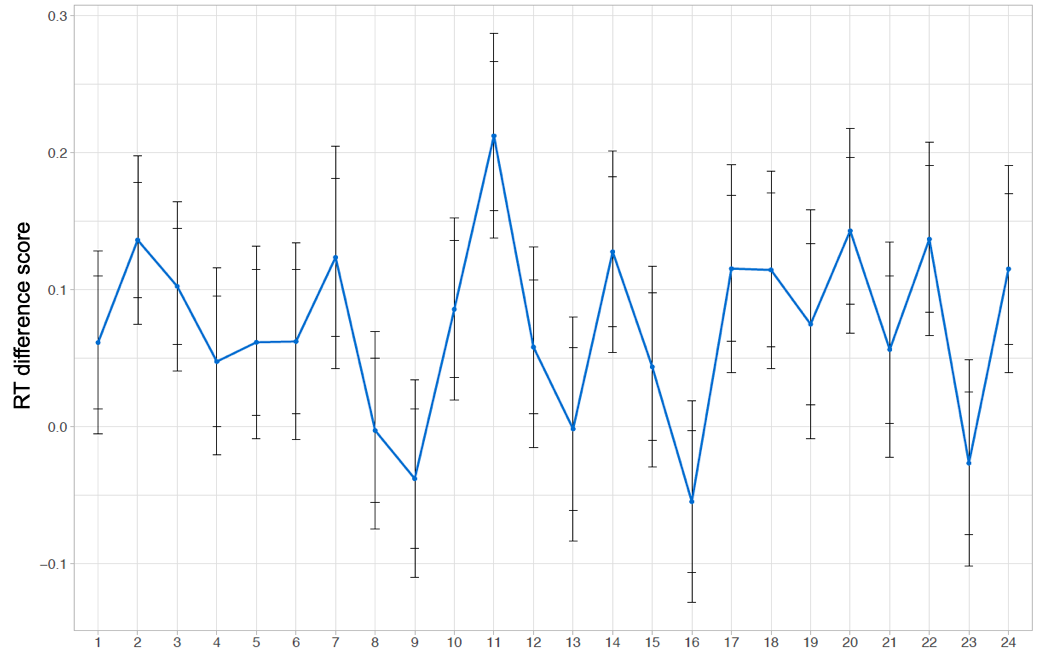


**Supplementary figure**: Descriptive results of the online RT data: difference score. Mean normalized RT to unpredictable element 1 minus mean normalized RT to predictable element 2, plotted per repetition of triplets during the experiment (see section 3.1.2 of the manuscript).
